# Event-related potential data from a guess the number brain-computer interface experiment on school children

**DOI:** 10.1038/sdata.2016.121

**Published:** 2017-03-28

**Authors:** R. Mouček, L. Vařeka, T. Prokop, J. Štěbeták, P. Brůha

**Affiliations:** 1 NTIS—New Technologies for the Information Society, Faculty of Applied Sciences, University of West Bohemia, Univerzitní 8, Plzeň 306 14, Czech Republic

**Keywords:** Electroencephalography - EEG, Neurophysiology, Cognitive neuroscience, Biomedical engineering

## Abstract

Guess the number is a simple P300-based brain-computer interface experiment. Its aim is to ask the measured participant to pick a number between 1 and 9. Then, he or she is exposed to corresponding visual stimuli and experimenters try to guess the number thought while they are observing event-related potential waveforms on-line. 250 school-age children participated in the experiments that were carried out in elementary and secondary schools in the Czech Republic. Electroencephalographic data from three EEG channels (Fz, Cz, Pz) and stimuli markers were stored. Additional metadata about the participants were collected (gender, age, laterality, the number thought by the participant, the guess of the experimenters, and various interesting additional information). Consequently, we offer the largest publicly available odd-ball paradigm collection of datasets to neuroscientific and brain-computer interface community.

## Background & Summary

Brain-computer interfaces (BCIs) allow communication without muscular activity based on brain signals measured with electroencephalography (EEG). The P300 component is an event-related potential elicited during the process of decision making. P300-based BCIs^1^ have been gaining attention in recent years and nowadays are considered one of the main BCI categories^2^. Compared to other BCI paradigms, P300 BCIs are relatively fast, effective for most users, straightforward, and require practically no training of the subjects^2^.

However, since BCIs typically rely on supervised classification, a substantial amount of P300 data is necessary for their training. Continuous EEG data are relatively common in data publications, e.g., (ref. 3). Unfortunately, data sharing is rare in P300-related publications. There are some publicly available P300 datasets, e.g., a benchmark P300 speller dataset from the BCI Competition 2003 (ref. 4). In ref. 5, the authors describe off-line analysis of P300 data for a simple BCI system. In addition, the authors offer the related data for sharing^5^. Both offered P300 datasets were obtained using short inter-stimulus intervals, 100 and 400 ms, respectively. The data are stored in various inner Matlab structures and the description of related metadata is limited. We have already published a smaller collection of P300 datasets based on a simple LED-based protocol^6^. With this exception, to the best knowledge of the authors, there is no publicly available database that offers P300 data for subsequent analysis while including metadata in a format widely accepted by the neuroscience community. Consequently, researchers in this area often use their own data. This limits the opportunity to reasonably compare different studies.

The aim of this article is to describe a large collection of P300 datasets and provide it to the scientific community.

The P300 data contained in the presented P300 collection of datasets were collected during the ‘Guess the number’ experiment. This experiment, based on visual stimulation, was originally developed to demonstrate the benefits of using BCI to public. The participant in the experiment is asked to choose a number between 1 and 9 and concentrate on it (i.e., this number is the target stimulus). Then, the subject is exposed to visual stimuli that include numbers between 1 and 9 randomly appearing on the monitor. During the experiment, both EEG signal and stimuli markers are recorded. Concurrently, experimenters observe average event-related potential (ERP) waveforms for each number and try to guess the number thought. Their guess is finally verified when the participant is asked to reveal the thought number.

## Methods

### Environment

The experiments were carried out in elementary and secondary schools, mainly located in the Pilsen region, the Czech Republic, between autumn 2014 and spring 2015. The measurements were taken at the time of regular school hours, typically in the morning. Each experiment was performed in a classroom that was arranged for health entertaining and educating programme including Neurosky brain games, ECG monitoring, modeling of body muscles, etc. Unfortunately, the environment was usually quite noisy since many children and also many electrical devices were present in the room at the same time. However, in any case there were no people standing or moving behind the monitor or in the close proximity of the measured participant.

### Stimulation protocol

**Figure 1:**
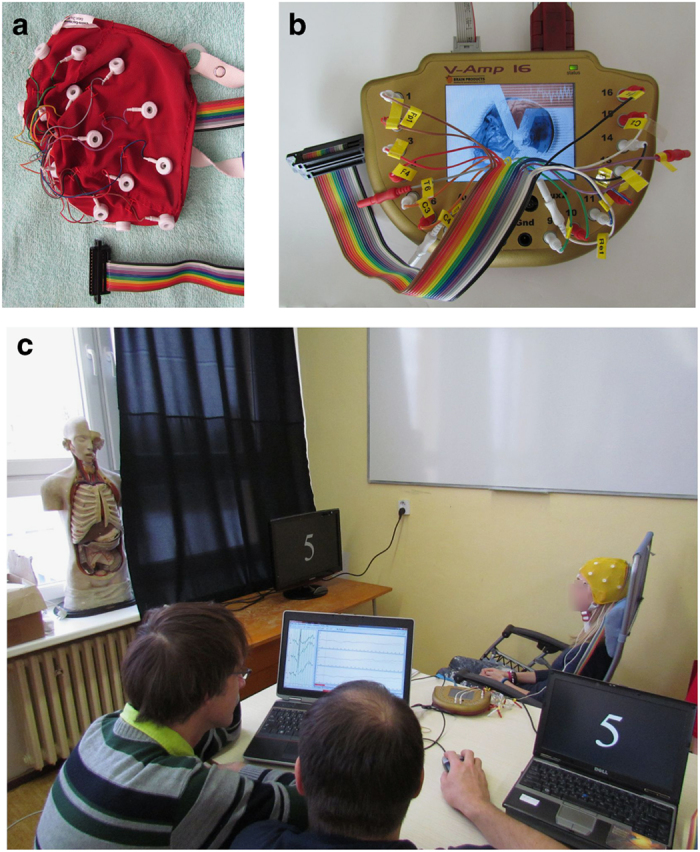
Experiment, hardware equipment. (**a**) The medium 10/20 EEG cap. (**b**) The BrainVision V-Amp amplifier. (**c**) Course of the experiment. Researchers are observing event-related potentials while these are averaged in the BrainVision Recorder. The notebook on the right is used to control the stimulation. The subject sitting in the chair is exposed to visual stimuli. The subject’s face has been blurred to protect his/her privacy.

The participants were stimulated with numbers between 1 and 9 flashing on the monitor in random order. The numbers were white on the black background as shown in Fig. 1c. The inter-stimulus interval was set to 1,500 ms.

### Hardware and software

A mobile EEG laboratory (equipment easy to unpack, operate, and pack again in the conditions described above) was transported to schools to perform experiments. More precisely, the following hardware devices were used: the BrainVision standard V-Amp amplifier (Fig. 1b), standard small or medium 10/20 EEG cap (Fig. 1a), standard reference, ground and EOG electrodes, monitor for presenting the numbers, and two notebooks necessary to run stimulation and recording software applications. To speed up the guessing task, only three electrodes, Fz, Cz and Pz, were active. The stimulation protocol was developed and run using the Presentation software tool produced by Neurobehavioral Systems, Inc. The BrainVision Recorder was used for recording and storing raw EEG data, metadata describing the raw data, and stimuli data. MATLAB, EEGLAB and ERPLAB were later used to validate the data.

### Participants and experimenters

The participants were school-age children and teenagers (aged between 7 and 17; average age 12.9), 138 males and 112 females. All participants and their parents were informed about the programme of the day and the experiments carried out. All participants took part in the experiment voluntarily. Many of them took part in more brain experiments during the day. The gender, age, and laterality of the participants were collected, it means that no personal or sensitive data such as names, birth dates or identifying physical symptoms were asked or recorded. Experiments, which were conducted in one day, were carried out in the same place in the same classroom.

There were usually three experimenters present. The first experimenter, a health-care professional in EEG, was responsible for preparing the participant for the experiment (by applying the EEG cap and electrodes) and explaining them the goal of the experiment and behavior rules that are necessary to follow during the experiment. This experimenter was also responsible for replacing the cap and electrodes after the end of the experiment. The second experimenter was mainly responsible for correct functioning of the used hardware and software infrastructure. The third experimenter spent most of his time explaining the nature of the experiment to the participant and to other onlookers. All experimenters usually participated in the main task—guessing the number.

### Data acquisition

Before starting the experiment itself the participants were informed about the goal of the experiment, course of the experiment, and used equipment. Each participant was familiarized with basic behavioral rules, asked to sit comfortably, pay attention to the stimulation, not to move, and limit their eye blinking. To increase alertness, the participants were instructed to silently count the total number of target stimuli presented on the monitor. During the experiment the participants were sitting approximately 1.5 m in front of the monitor for as long as needed (approximately 10 min on average). Other children observing the experiment were asked not to enter into the field of view of the participant and not to disturb him/her in any other way.

Then the participant was technically prepared for the experiment: an EEG cap was used depending on the size of the participant’s head, the reference electrode was placed on the bridge of the nose and the ground electrode was placed on the ear. The EOG electrode for observing eye movements was placed under the participant’s eye. The reference, ground, and EOG electrodes as well as the EEG cap were connected to the V-Amp amplifier. The impedances of all electrodes were checked and corrected if necessary. When the participant assured experimenters that he/she had understand all the circumstances of the experiment and selected a target number to concentrate on, the experiment was launched.

During the experiment the participant was regularly checked if he/she was following the rules. If the signal was damaged by eye blinking or other movement artifacts, the participant was asked to reduce these movements. However, there were several cases when the experiment was terminated prematurely because of a large number of artifacts or bad feelings (nausea, headache) of the participant. Normally the experiment was stopped at the time the experimenters decided to guess the number or assumed not having any chance to guess the number from the signal. If the experimenters were not successful in guessing the number, they usually asked the participant to continue in the experiment and tried the second or even the third guess. After finishing the experiment the experimenters showed and explained the participant his/her results including the P300 average waveforms. The explanation was always adjusted to the age of the participant.

## Data Records

### Data storage

The EEG/ERP Portal (EEGBase) (Data Citation 1) was used for storing the experimental data and metadata. It is a web application that serves not only for long-term storage of EEG/ERP experiments, but also for their annotation, management, and sharing. The stored data are protected by the system of user accounts and defined user roles (Reader, Experimenter, Group Administrator, and Supervisor). Individual users are grouped into self-managed groups. The user is required to create a personal account prior to uploading or downloading any experiment. Metadata are stored using metadata templates that reflect the odML terminologies^7^.

Although the EEG/ERP Portal has been developed and optimized as a data storage for human EEG data, it does not provide direct and permanent download links for individual datasets. Currently the EEG/ERP Portal also does not support DOI citations. Therefore, each dataset stored in the EEG/ERP Portal is mirrored in the Harvard Dataverse (Data Citations 2–251).

### Data organization

The data and metadata from 250 participants are stored in the EEG/ERP Portal and downloadable as ‘PROJECT DAYS P3 NUMBERS’ zip package (the procedure of getting this package is described in Section usage notes). Each dataset has its own folder that is further internally organized in the following way:

1. the experimental protocol (the files generated by the Presentation software) is located in the Scenario folder,
2. the experimental data and metadata stored in the BrainVision format (.eeg,.vhdr and .vmrk files) and the basic experimental metadata (.txt file) are located in the Data folder.
3. P3Numbers_yyyymmdd_gender_age_id.eeg is a binary file containing raw EEG data,
4. P3Numbers_yyyymmdd_gender_age_id.vhdr is a text file containing metadata that describe raw EEG data stored in the corresponding.eeg file,
5. P3Numbers_yyyymmdd_gender_age_id.vmrk is a text file containing stimuli markers used in the experiment,
6. P3Numbers_yyyymmdd_gender_age_id.txt is a text file containing basic experimental metadata—gender, age, the number thought, first guessed number, second guessed number, third guessed number, laterality, and eventually any interesting additional information (the field named as ‘other’) collected on site (these metadata are presented separately because they did not meet fully the allowable content of EEG/ERP Portal metadata templates at the time when they were collected and stored).
7. The License agreement (Creative Commons Attribution Non Commercial 4.0) is located in the License folder.
8. The experimental metadata file (metadata.xml) contains a set of metadata (such as used hardware and software) describing the experimental conditions. It is stored in the root folder of each dataset and structured according to the portal metadata template used for data storing. It reflects the EEG/ERP Portal terminology restrictions applied to the metadata content at the time the metadata were collected and stored (These metadata restrictions are no more applied; currently all experimental metadata could be stored in one file only).

While all the described files are organized in a hierarchical folder structure within a .zip package when they are downloaded from the EEG/ERP Portal, this hierarchical structure is not applied to the replicated data in the Harvard Dataverse repository (Data Citations 2–251). Instead, the files are organized in the plain structure there.

## Technical Validation

**Figure 2:**
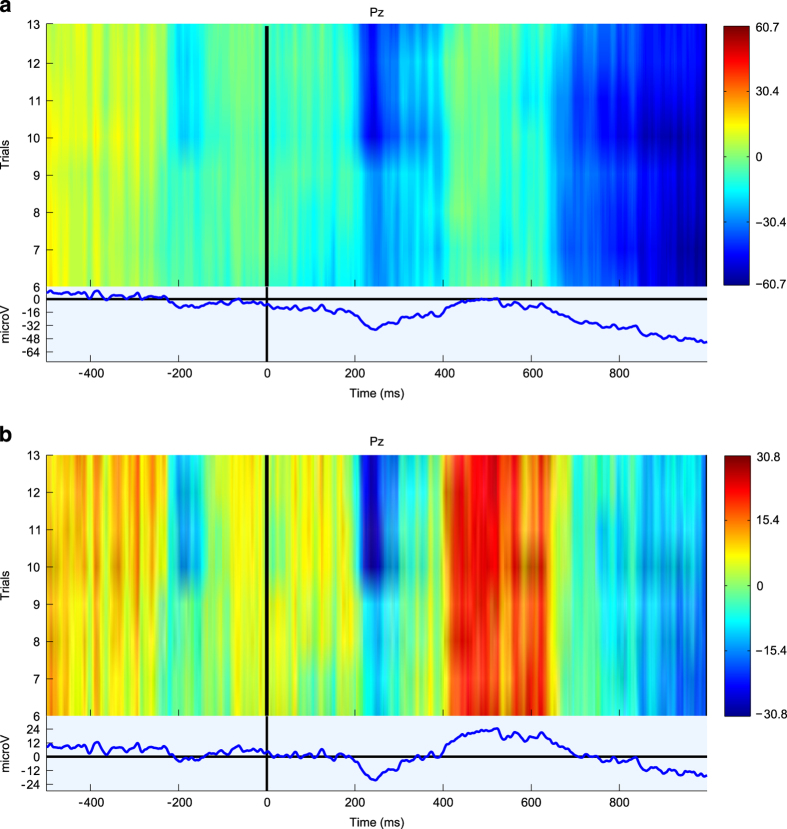
Averaged Pz channel epochs of the target stimulus. (**a**) Averaged Pz channel epochs of the target stimulus of the experiment with ID 341 (data file P3Numbers_20150618_f_10_001.eeg). Epochs were extracted in the interval −500 ms to 1,000 ms relative to stimulus onset. Subsequently a baseline acquired from the −500 ms to 0 ms interval before the stimulus was subtracted from each epoch. The declining signal trend is clearly visible on the plot even after baseline correction. The trials above the chart represent moving averages with the smoothing window of length 10. The original data consist of 17 trials. The scale on the right side is in microvolts. (**b**) The same baseline corrected epochs averaged after high pass filtering with 0.5 Hz cut-off frequency. The declining signal trend was removed by the high pass filter.

All data were saved in a raw form. It means that the preprocessing methods (filtering, baseline correction, artifacts rejection) applied to the data during experimental sessions (to visualize and analyze them) were not applied to the stored data. The quality of datasets varies because all measurements were performed outside the laboratory. EEG signal of most datasets shows a declining signal trend. Because of that we tested the hardware amplifier for possible defects and took measures to eliminate sources of interference in classrooms as much as possible. We believe that the declining signal trend was caused by outside interference we could no longer influence (see Fig. 2). This issue can be easily handled by applying high pass filtering (with cutoff frequency e.g., 0.5 Hz). Most data were first stored on a laptop that was running on battery power. However, in the case of low battery power (only during the days when many experiments were carried out) it was necessary to switch the power source to grid. Then the data contain 50 Hz interference that can be also removed by filtering.

The technical validation of each dataset was performed separately. Two different parameters were considered:

- The rate of eye-blinking artifacts in ERP epochs. Eye-blinks severely distort the EEG signal and reduce the usability of datasets. The percentage of epochs damaged by eye blinks was calculated using a combination method described in^8^ for each experiment separately. The combination method iterates over all baseline-corrected ERP epochs. For eye-blinks detection, it uses a combined threshold factoring in maximum absolute value of amplitude and a correlation with a sample eye-blink. The results for each dataset are available in Table 1 (available online only).
- It was evaluated if the number thought was correctly guessed by the experimenters or not. The experiments with successful guesses are typically associated with a larger amplitude of the P300 component. The results for each dataset are available in Table 1 (available online only).

## Usage Notes

**Figure 3:**
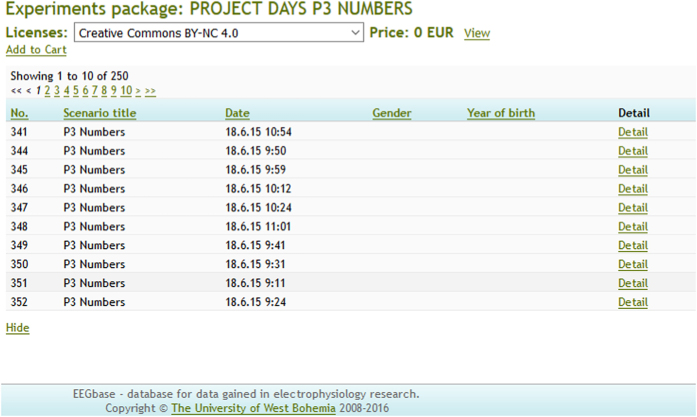
EEG/ERP Portal (EEGBase)—list of experiments. List of experiments in the package ‘PROJECT DAYS P3 NUMBERS’ (only the first ten experiments are showed) and the ‘Add to cart’ link.

The experimental data and metadata can be downloaded from the EEG/ERP Portal (Data Citation 1) according to the following procedure. Any user has to be registered first. When the registration form is completed, a confirmation e-mail is sent to the user. Then the user is requested to click on the confirmation link contained in the confirmation e-mail. After successful login a personalized user’s homepage including an overview of user’s experiments, scenarios, research group memberships, etc. is displayed. In order to see publicly offered experiments and find the package named ‘PROJECT DAYS P3 NUMBERS’ the user selects the Experiments section from the main menu appearing at the top of the homepage. When the Experiment section is loaded, the user selects the package ‘PROJECT DAYS P3 NUMBERS’, chooses the license under which he/she wants to use the data (Creative Commons BY-NC) and clicks on the ‘Add to cart’ link (see Fig. 3).

**Figure 4:**
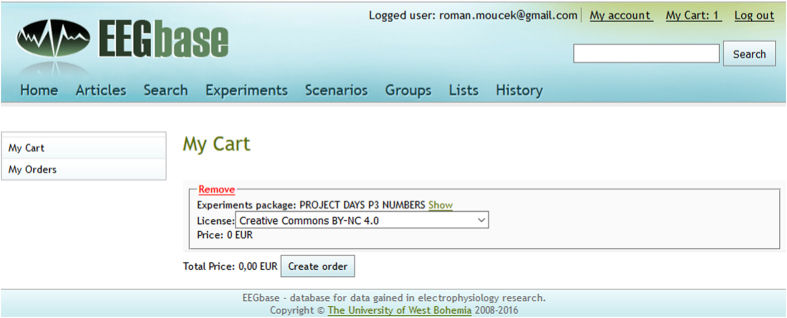
EEG/ERP Portal (EEGBase)—Content of the cart. Content of the cart that is available under the selected license.

When the package is added into the cart, the user is requested to click on the ‘My cart’ link at the top of the page. The content of the cart is shown (Fig. 4). The experiments in the ‘PROJECT DAYS P3 NUMBERS’ package are available under the selected license. When the user finishes the order (by clicking on the ‘Create order’ button), the package is formally available for downloading (by clicking on the ‘Download’ link). Then the user confirms his/her selection of the experiments within the package and clicks on the ‘Create package’ button to create a.zip package (PROJECT_DAYS_P3_NUMBERS.zip). Since the data are quite large, the progress bar indicates the portion of the package that has been already created. When the package is created, it can be finally downloaded by clicking on the ‘Download’ link.

The ordered (purchased) package could be re-downloaded at any time in the Experiment section by clicking on the ‘Download’ link that appears instead of the ‘Add to cart’ link within the package.

Since the data were stored in the BrainVision (BV) format^9^, appropriate software tools have to be used to read and further process the data. EEGLab, an open-source Matlab toolbox for EEG signal processing^10^, is one of the preferred options. It is necessary to download the BVA-io plugin (available at http://sccn.ucsd.edu/wiki/EEGLAB_Extensions_and_plug-ins) in order to easily import the data stored in the BV format into EEGLab. Another option is to use the EEGLoader library (available at https://github.com/stebjan/eegloader) that provides a simple interface for reading the BV format.

## Additional Information

**How to cite this article:** Mouček, R. *et al.* Event-related potential data from a guess the number brain-computer interface experiment on school children. *Sci. Data* 4:160121 doi: 10.1038/sdata.2016.121 (2017).

**Publisher’s note:** Springer Nature remains neutral with regard to jurisdictional claims in published maps and institutional affiliations.

## Supplementary Material



## Figures and Tables

**Table 1:** The technical validation of datasets

**Sample Name**	**Sex**	**Age [Year]**	**The number thought**	**Artefacts [%]**	**Guessed by Expert**
P3Numbers_20150618_f_10_001	female	10	1	12.5±17.265	yes
P3Numbers_20150618_f_11_001	female	11	5	22.599±17.265	yes
P3Numbers_20150618_f_11_002	female	11	4	64.103±17.265	no
P3Numbers_20150618_f_11_003	female	11	3	6.395±17.265	no
P3Numbers_20150618_f_11_004	female	11	3	10.377±17.265	yes
P3Numbers_20150618_f_11_005	female	11	4	14.07±17.265	yes
P3Numbers_20150618_f_12_001	female	12	1	25±17.265	no
P3Numbers_20150618_m_11_001	male	11	2	45.291±17.265	yes
P3Numbers_20150618_m_12_001	male	12	1	49.587±17.265	no
P3Numbers_20150618_m_12_002	male	12	4	26.923±17.265	yes
P3Numbers_20150414_f_12_001	female	12	8	6.897±17.265	yes
P3Numbers_20150414_f_13_001	female	13	6	20.37±17.265	no
P3Numbers_20150414_f_13_002	female	13	4	2.139±17.265	yes
P3Numbers_20150414_f_13_003	female	13	4	10.345±17.265	yes
P3Numbers_20150414_m_13_001	male	13	6	15.789±17.265	yes
P3Numbers_20150414_m_13_002	male	13	7	17.949±17.265	yes
P3Numbers_20150414_m_13_003	male	13	7	63.75±17.265	no
P3Numbers_20150618_f_11_006	female	11	5	71.774±17.265	no
P3Numbers_20150618_f_11_007	female	11	3	14.563±17.265	yes
P3Numbers_20150618_m_11_002	male	11	5	12.162±17.265	no
P3Numbers_20150414_f_11_001	female	11	1	32.292±17.265	yes
P3Numbers_20150414_f_11_002	female	11	8	19.008±17.265	yes
P3Numbers_20150414_f_13_004	female	13	5	6.452±17.265	yes
P3Numbers_20150414_f_14_001	female	14	2	26.378±17.265	yes
P3Numbers_20150414_f_14_002	female	14	4	17.241±17.265	yes
P3Numbers_20150414_f_14_003	female	14	9	15.217±17.265	yes
P3Numbers_20150414_f_15_001	female	15	5	12.637±17.265	yes
P3Numbers_20150414_m_13_004	male	13	3	11.321±17.265	yes
P3Numbers_20150414_m_14_001	male	14	8	12.987±17.265	yes
P3Numbers_20150414_m_14_002	male	14	-	61.905±17.265	yes
P3Numbers_20150414_m_15_001	male	15	3	35.082±17.265	no
P3Numbers_20150414_m_14_003	male	14	8	18.072±17.265	yes
P3Numbers_20150414_f_15_004	female	15	6	31.25±17.265	yes
P3Numbers_20150414_f_15_003	female	15	7	26.214±17.265	yes
P3Numbers_20150414_f_15_002	female	15	3	19.527±17.265	yes
P3Numbers_20150414_f_14_004	female	14	5	11.915±17.265	no
P3Numbers_20141023_m_12_002	male	12	1	0.515±17.265	yes
P3Numbers_20141023_m_12_001	male	12	8	0±17.265	yes
P3Numbers_20141023_f_14_001	female	14	1	0±17.265	yes
P3Numbers_20141023_f_11_001	female	11	3	0±17.265	yes
P3Numbers_20141023_m_15_003	male	15	1	0±17.265	yes
P3Numbers_20141023_f_11_002	female	11	7	0±17.265	yes
P3Numbers_20141023_f_12_001	female	12	3	0±17.265	yes
P3Numbers_20141023_f_11_003	female	11	4	0±17.265	no
P3Numbers_20141023_f_12_002	female	12	1	0±17.265	yes
P3Numbers_20141023_f_13_001	female	13	6	0±17.265	yes
P3Numbers_20141023_f_14_002	female	14	3	0.912±17.265	no
P3Numbers_20141023_f_14_003	female	14	2	0±17.265	no
P3Numbers_20141023_f_15_001	female	15	2	0±17.265	no
P3Numbers_20141023_m_13_001	male	13	3	0±17.265	yes
P3Numbers_20141023_m_14_001	male	14	3	0±17.265	yes
P3Numbers_20141023_m_14_002	male	14	2	4.29±17.265	no
P3Numbers_20141023_m_15_001	male	15	6	0.316±17.265	no
P3Numbers_20141023_m_15_002	male	15	9	0±17.265	yes
P3Numbers_20150224_f_09_001	female	9	5	18.182±17.265	no
P3Numbers_20150224_f_10_001	female	10	8	18.929±17.265	no
P3Numbers_20150224_f_11_001	female	11	7	2.236±17.265	no
P3Numbers_20141124_f_12_001	female	13	3	11.18±17.265	no
P3Numbers_20141124_f_12_002	female	12	4	6.154±17.265	yes
P3Numbers_20141124_f_13_001	female	13	3	5.573±17.265	no
P3Numbers_20141124_f_13_002	female	13	8	11.404±17.265	yes
P3Numbers_20141124_f_14_001	female	14	3	58.369±17.265	yes
P3Numbers_20141124_f_14_002	female	14	6	35.556±17.265	yes
P3Numbers_20141124_f_14_003	female	14	9	3.704±17.265	yes
P3Numbers_20141124_f_14_004	female	14	1	8.333±17.265	yes
P3Numbers_20141124_f_15_001	female	15	9	23.574±17.265	yes
P3Numbers_20141124_f_17_001	female	17	3	17±17.265	yes
P3Numbers_20141124_f_17_002	female	17	4	42.222±17.265	yes
P3Numbers_20141124_m_12_001	male	12	4	54.237±17.265	yes
P3Numbers_20141124_m_12_002	male	12	7	19.298±17.265	yes
P3Numbers_20141124_m_13_001	male	13	6	10.425±17.265	yes
P3Numbers_20141124_m_14_001	male	14	1	27.119±17.265	yes
P3Numbers_20141124_m_14_002	male	14	5	31.788±17.265	yes
P3Numbers_20141124_m_14_003	male	14	8	11.881±17.265	yes
P3Numbers_20141124_m_14_004	male	14	2	39.427±17.265	no
P3Numbers_20141124_m_15_001	male	15	9	27.82±17.265	yes
P3Numbers_20141124_m_17_001	male	17	2	20±17.265	no
P3Numbers_20141204_f_10_001	female	10	1	14.103±17.265	yes
P3Numbers_20141204_f_11_001	female	11	3	19.38±17.265	yes
P3Numbers_20141204_f_11_002	female	11	3	41.155±17.265	no
P3Numbers_20141204_f_11_003	female	11	1	33.889±17.265	yes
P3Numbers_20141204_f_11_004	female	11	6	9.091±17.265	yes
P3Numbers_20141204_f_11_005	female	11	7	26.452±17.265	no
P3Numbers_20141204_f_11_006	female	11	5	46.025±17.265	no
P3Numbers_20141204_f_11_007	female	11	7	20.122±17.265	yes
P3Numbers_20141204_f_12_001	female	12	3	27.815±17.265	no
P3Numbers_20141204_f_12_002	female	12	5	46.512±17.265	yes
P3Numbers_20141204_f_12_003	female	12	5	64.516±17.265	no
P3Numbers_20141204_f_13_001	female	13	7	38.095±17.265	yes
P3Numbers_20141204_f_13_002	female	13	2	27.723±17.265	yes
P3Numbers_20141204_f_14_001	female	14	5	8.333±17.265	no
P3Numbers_20141204_f_14_002	female	14	5	49.375±17.265	no
P3Numbers_20141204_f_15_001	female	15	1	27.848±17.265	yes
P3Numbers_20141204_m_08_001	male	8	2	18.327±17.265	yes
P3Numbers_20141204_m_11_001	male	11	4	46.486±17.265	no
P3Numbers_20141204_m_11_002	male	11	2	65.193±17.265	yes
P3Numbers_20141204_m_12_001	male	12	5	5.825±17.265	yes
P3Numbers_20141204_m_12_002	male	12	2	61.039±17.265	yes
P3Numbers_20141204_m_13_001	male	13	7	46.939±17.265	yes
P3Numbers_20141204_m_14_001	male	14	5	15.638±17.265	no
P3Numbers_20141204_m_14_002	male	14	3	59.574±17.265	no
P3Numbers_20150224_f_11_002	female	11	2	25.49±17.265	yes
P3Numbers_20150224_f_14_001	female	14	7	57.54±17.265	no
P3Numbers_20150224_f_15_001	female	15	4	7.19±17.265	yes
P3Numbers_20150224_m_11_001	male	11	7	52.874±17.265	yes
P3Numbers_20150224_m_11_002	male	11	7	31.731±17.265	yes
P3Numbers_20150224_m_11_003	male	11	4	51.399±17.265	yes
P3Numbers_20150224_m_11_004	male	11	7	32.416±17.265	no
P3Numbers_20150224_m_12_001	male	12	3	22.33±17.265	yes
P3Numbers_20150224_m_12_002	male	12	5	17.46±17.265	yes
P3Numbers_20150224_m_12_003	male	12	4	46.667±17.265	yes
P3Numbers_20150224_m_13_001	male	13	6	12.037±17.265	yes
P3Numbers_20150224_m_13_002	male	13	4	52.83±17.265	yes
P3Numbers_20150224_m_14_001	male	14	3	15.574±17.265	no
P3Numbers_20150224_m_15_001	male	15	3	53.226±17.265	no
P3Numbers_20150224_m_15_002	male	15	3	13.621±17.265	no
P3Numbers_20150224_m_15_003	male	15	3	78.829±17.265	yes
P3Numbers_20150224_m_15_004	male	15	3	11.321±17.265	yes
P3Numbers_20150507_f_14_001	female	14	1	35.621±17.265	yes
P3Numbers_20150507_f_14_002	female	14	3	12.15±17.265	yes
P3Numbers_20150507_f_14_003	female	14	6	22.807±17.265	yes
P3Numbers_20150507_f_14_004	female	14	2	41.262±17.265	yes
P3Numbers_20150507_f_14_005	female	14	4	17.969±17.265	yes
P3Numbers_20150507_m_13_001	male	13	3	16.556±17.265	yes
P3Numbers_20150507_m_14_001	male	14	7	8.772±17.265	yes
P3Numbers_20150507_m_14_002	male	14	3	10.526±17.265	yes
P3Numbers_20150507_m_14_003	male	14	2	9.804±17.265	no
P3Numbers_20150507_m_14_004	male	14	9	14.729±17.265	yes
P3Numbers_20150507_m_14_005	male	14	6	2.198±17.265	no
P3Numbers_20150507_m_14_006	male	14	7	16.092±17.265	yes
P3Numbers_20150507_m_14_007	male	14	8	19.355±17.265	yes
P3Numbers_20150507_m_15_001	male	15	5	7.808±17.265	no
P3Numbers_20150507_m_16_001	male	16	6	25±17.265	yes
P3Numbers_20150107_f_13_001	female	13	3	23.967±17.265	yes
P3Numbers_20150107_f_14_001	female	14	5	11.268±17.265	yes
P3Numbers_20150107_f_14_002	female	14	3	18.713±17.265	yes
P3Numbers_20150107_f_14_003	female	14	2	2.941±17.265	yes
P3Numbers_20150107_f_14_004	female	14	3	22.656±17.265	yes
P3Numbers_20150107_f_14_005	female	14	8	30.769±17.265	yes
P3Numbers_20150107_f_14_006	female	14	2	74.818±17.265	yes
P3Numbers_20150107_f_14_007	female	14	2	33.333±17.265	yes
P3Numbers_20150107_f_15_001	female	15	8	43.293±17.265	yes
P3Numbers_20150107_m_14_001	male	14	5	12.637±17.265	yes
P3Numbers_20150107_m_14_003	male	14	1	22.472±17.265	yes
P3Numbers_20150107_m_14_002	male	14	5	29.63±17.265	yes
P3Numbers_20150107_m_14_004	male	14	7	28.4±17.265	no
P3Numbers_20150107_m_14_005	male	14	4	49.194±17.265	yes
P3Numbers_20150107_m_15_001	male	15	8	27.041±17.265	yes
P3Numbers_20150314_m_12_001	male	12	7	23.265±17.265	no
P3Numbers_20150203_m_15_001	male	15	7	34.185±17.265	yes
P3Numbers_20150203_m_16_001	male	16	3	32.963±17.265	no
P3Numbers_20150203_m_17_001	male	17	7	21.3±17.265	no
P3Numbers_20150203_m_17_002	male	17	1	42.213±17.265	no
P3Numbers_20150203_m_17_003	male	17	4	8.214±17.265	no
P3Numbers_20150203_m_17_004	male	17	8	40.85±17.265	yes
P3Numbers_20150203_m_17_005	male	17	5	17.297±17.265	yes
P3Numbers_20150203_m_17_006	male	17	6	39.42±17.265	no
P3Numbers_20150126_f_09_001	female	9	6	30.723±17.265	yes
P3Numbers_20150126_f_09_002	female	9	5	31.579±17.265	yes
P3Numbers_20150126_f_10_001	female	10	3	13.953±17.265	yes
P3Numbers_20150126_f_11_001	female	11	7	45.196±17.265	yes
P3Numbers_20150126_f_12_001	female	12	7	12.342±17.265	yes
P3Numbers_20150126_f_12_002	female	12	1	37.778±17.265	yes
P3Numbers_20150126_f_12_003	female	12	7	7.229±17.265	yes
P3Numbers_20150126_f_12_004	female	12	4	44.177±17.265	yes
P3Numbers_20150126_f_13_001	female	13	5	32.899±17.265	no
P3Numbers_20150126_f_14_001	female	14	5	32.927±17.265	no
P3Numbers_20150126_f_14_001	female	14	5	32.927±17.265	no
P3Numbers_20150126_m_07_001	male	7	9	67.083±17.265	no
P3Numbers_20150126_m_09_001	male	9	8	28.125±17.265	no
P3Numbers_20150126_m_12_001	male	12	5	45.161±17.265	yes
P3Numbers_20150126_m_12_002	male	12	7	59.33±17.265	no
P3Numbers_20150126_m_12_003	male	12	6	30.337±17.265	no
P3Numbers_20150126_m_12_004	male	12	5	61.429±17.265	no
P3Numbers_20150126_m_13_001	male	13	1	21.778±17.265	yes
P3Numbers_20150126_m_13_001	male	13	1	21.778±17.265	yes
P3Numbers_20150126_m_13_002	male	13	4	48.399±17.265	yes
P3Numbers_20150126_m_15_001	male	15	3	9.412±17.265	yes
P3Numbers_20150126_m_15_002	male	15	3	25±17.265	no
P3Numbers_20150126_m_15_003	male	15	6	58.759±17.265	yes
P3Numbers_20150126_m_15_004	male	15	5	29.57±17.265	no
P3Numbers_20150204_f_11_001	female	11	5	29.771±17.265	yes
P3Numbers_20150204_f_11_002	female	11	6	11.05±17.265	no
P3Numbers_20150204_f_12_001	female	12	1	4.839±17.265	yes
P3Numbers_20150204_f_12_002	female	12	2	10.152±17.265	yes
P3Numbers_20150204_f_12_003	female	12	2	27.907±17.265	no
P3Numbers_20150204_f_13_001	female	13	2	13.74±17.265	yes
P3Numbers_20150204_f_13_002	female	13	6	31.373±17.265	yes
P3Numbers_20150204_f_13_003	female	13	8	24.336±17.265	yes
P3Numbers_20150204_m_12_001	male	12	4	29.301±17.265	no
P3Numbers_20150204_m_12_002	male	12	6	29.534±17.265	no
P3Numbers_20150204_m_12_003	male	12	6	4.457±17.265	no
P3Numbers_20150204_m_12_004	male	12	3	29.47±17.265	yes
P3Numbers_20150204_m_12_005	male	12	3	30.859±17.265	yes
P3Numbers_20150204_m_12_006	male	12	3	30.07±17.265	yes
P3Numbers_20150204_m_13_001	male	13	7	49.761±17.265	no
P3Numbers_20150204_m_13_002	male	13	9	33.333±17.265	yes
P3Numbers_20150204_m_13_003	male	13	2	25.368±17.265	no
P3Numbers_20150204_m_13_004	male	13	5	55.556±17.265	no
P3Numbers_20150204_m_13_005	male	13	2	12.766±17.265	no
P3Numbers_20150204_m_14_001	male	14	2	8.421±17.265	no
P3Numbers_20150326_f_13_001	female	13	7	7.042±17.265	yes
P3Numbers_20150326_f_13_002	female	13	8	44.978±17.265	no
P3Numbers_20150326_f_15_001	female	15	2	17.162±17.265	no
P3Numbers_20150326_m_14_001	male	14	3	5.164±17.265	yes
P3Numbers_20150326_m_14_002	male	14	9	21.809±17.265	yes
P3Numbers_20150326_m_15_001	male	15	7	5.106±17.265	yes
P3Numbers_20150326_m_15_002	male	15	4	6.051±17.265	no
P3Numbers_20150326_m_15_003	male	15	7	35.361±17.265	yes
P3Numbers_20150326_m_15_004	male	15	5	53.175±17.265	no
P3Numbers_20150326_m_16_001	male	16	2	5.389±17.265	yes
P3Numbers_20150514_f_09_001	female	9	4	26.087±17.265	no
P3Numbers_20150514_f_09_002	female	9	9	12.081±17.265	yes
P3Numbers_20150514_f_10_001	female	10	2	12.876±17.265	no
P3Numbers_20150514_f_10_002	female	10	7	8.485±17.265	yes
P3Numbers_20150514_f_10_003	female	10	9	6.41±17.265	yes
P3Numbers_20150514_m_09_001	male	9	5	16.923±17.265	yes
P3Numbers_20150514_m_10_001	male	10	6	35.276±17.265	no
P3Numbers_20150514_m_10_002	male	10	2	19.068±17.265	no
P3Numbers_20150514_m_11_001	male	11	9	40.672±17.265	no
P3Numbers_20150514_m_11_002	male	11	5	18.902±17.265	yes
P3Numbers_20150514_m_12_001	male	12	3	9.459±17.265	yes
P3Numbers_20150514_m_12_002	male	12	3	14.226±17.265	no
P3Numbers_20150514_m_12_003	male	12	7	31.373±17.265	no
P3Numbers_20150514_m_13_001	male	13	8	26.066±17.265	yes
P3Numbers_20150514_m_13_002	male	13	8	37.545±17.265	no
P3Numbers_20150514_m_13_003	male	13	6	10.084±17.265	yes
P3Numbers_20150514_m_13_004	male	13	7	26.172±17.265	no
P3Numbers_20150514_m_13_005	male	13	7	17.763±17.265	no
P3Numbers_20150514_m_13_006	male	13	6	22.656±17.265	no
P3Numbers_20150514_m_13_007	male	13	9	20.574±17.265	yes
P3Numbers_20150526_f_13_001	female	13	7	15.472±17.265	yes
P3Numbers_20150526_f_13_002	female	13	8	26.144±17.265	yes
P3Numbers_20150526_f_13_003	female	13	7	4.286±17.265	yes
P3Numbers_20150526_f_14_001	female	14	3	32.237±17.265	yes
P3Numbers_20150526_m_11_001	male	11	3	31.667±17.265	yes
P3Numbers_20150526_m_12_001	male	12	7	23.944±17.265	no
P3Numbers_20150526_m_12_002	male	12	7	19.118±17.265	no
P3Numbers_20150526_m_12_003	male	12	4	11.864±17.265	yes
P3Numbers_20150526_m_12_004	male	12	6	33.333±17.265	no
P3Numbers_20150526_m_13_001	male	13	3	25±17.265	no
P3Numbers_20150526_m_13_002	male	13	9	45.221±17.265	yes
P3Numbers_20150526_m_13_003	male	13	3	43.617±17.265	yes
P3Numbers_20150526_m_13_004	male	13	9	16.667±17.265	yes
P3Numbers_20150526_m_13_005	male	13	5	26.241±17.265	no
P3Numbers_20150526_m_14_001	male	14	7	3.704±17.265	yes
P3Numbers_20150526_m_14_002	male	14	6	32.824±17.265	yes
P3Numbers_20150526_m_14_003	male	14	5	13.043±17.265	yes
P3Numbers_20150526_m_14_004	male	14	3	24.409±17.265	no
P3Numbers_20150526_m_14_005	male	14	6	11.556±17.265	no
For each dataset, the percentage of epochs damaged by eye-blinking artifacts was calculated (in the Artifacts column). If the experimenters were able to guess the number thought, the corresponding value in the Guessed column is 'yes'.					
